# Ecophysiological and nutritional characterisation of two morphotypes of *Cakile maritima* subsp. *maritima* Scop. from Puglia region, Southern Italy

**DOI:** 10.3389/fpls.2024.1397852

**Published:** 2024-06-14

**Authors:** Giulia Conversa, Lucia Botticella, Corrado Lazzizera, Anna Bonasia, Antonio Elia

**Affiliations:** Department of Agriculture, Food, Natural Resources and Engineering (DAFNE), University of Foggia, Foggia, Italy

**Keywords:** sea rocket, halophyte, leaf gas exchanges, chlorophyll fluorescence, phenols, glucosinulates, iodine, vitamin C

## Abstract

*Cakile maritima* subsp. *maritima* Scop. (sea rocket) is a succulent halophyte with significant potential as a nutritious food source, being rich in essential nutrients such as vitamins, minerals, and antioxidants. This annual species exhibits two distinct leaf morphotypes: entire lamina (EL) and pinnatifid lamina (PL). Our understanding of their ecophysiological and nutritional profiles is still limited. The present study investigated the wild EL and PL sea rocket plants from southern Italy during their vegetative stage. The bio-morphological traits (leaf mass area-LMA, dry matter and chlorophyll concentrations), main inorganic ions, key antioxidants (carotenoids, anthocyanins, phenols, flavonoids, glucosinolates, vitamin C as ascorbic and dehydroascorbic acid), and antioxidant activity (by FRAP, DPPH, ABTS assays) were analyzed. Additionally, photosynthetic gas exchange and chlorophyll fluorescence were measured. PL plants showed thicker leaves (higher LMA) and greater accumulation of photo-protective pigments (carotenoids and anthocyanins), despite similar chlorophyll levels. The PL plants also demonstrated higher photosynthetic activity, transpiration rates, and stomatal conductance, with reduced non-photochemical quenching. The EL morphotype had higher cation (K, Mg, Ca, Na) and vitamin C (135.3 mg 100 g^-1^ FW) concentrations, while no significant disparities were observed between the morphotypes in phenolic concentration (208.5 mg g.a.e. 100 g^-1^ FW), flavonoids (71.5 mg q.e. 100 g^-1^ FW), or glucosinolates (61 mg g^-1^ FW). Interestingly, while the EL type had higher vitamin C, the PL morphotype showed superior antioxidant activity (FRAP, DPPH) and seems to be better adapted to water/nutrient scarcity typical of southern Italy. Both morphotypes offer potential as high-nutritional foods, however, future research should investigate the genotype-specific production of antioxidant compounds in EL and PL plants in response to environmental stresses, including salinity for potential exploitation as a new crop.

## 1 Introduction

Sea rocket, *Cakile maritima* subsp. *maritima* Scop., is a annual succulent halophyte belonging to the Brassicaceae family. Abundantly found along the sandy coasts of the Atlantic and Mediterranean regions, particularly on strandlines and associated foredunes, this plant exhibits branched, prostrate, or ascending stems that are initially green and succulent, but become fibrous with maturity, adopting a bush-like appearance. Inflorescences, in the form of dense racemes, terminate both the main stem and branches. The fleshy leaves frequently have marked phenotypic variation between populations, ranging from entire obovate to deeply pinnately lobed lamina. In Italy, specifically in the regions of Tuscany (Ciccarelli et al., 2010) and Puglia (Conversa, personal communication), evidence from plants grown in the same environment confirms the presence of two morphotypes distinguished by entire or pinnatifid leaves.

Traditionally, tender leaves, along with green stems, flowers and fruits are consumed fresh in salads or cooked, however, due to their bitter taste it is usually used as a food flavouring ingredient. Powdered roots of *C. maritima* can also be utilized in bread-making (Guil-Guerrero et al., 1999; Merchaoui et al., 2016; Agudelo et al., 2021).

In the Puglia region, sea rocket proliferates along the extended sandy coasts, and its culinary use is limited or sporadic, except for a specific area in the northern part of the region, where traditional dishes incorporating sea rocket are still prepared (Accogli et al., 2023) and are gaining interest for gourmet preparations. In this area, near the village of Margherita di Savoia and the city of Barletta (BT province), along the natural habitat represented by a sandy dunal cordon, fields, called ‘*arenili*’ extend for 30 km alongside the sea (Conversa et al., 2020a). Here, sea rocket has become a nuisance weed for onion and carrot crops (**Supplementary Figure 1**) and it can be easily harvested by locals.

Sea rocket thrives in harsh coastal environments, characterized by a combination of environmental stressors, to which the species has developed tolerance. These stressors include high temperatures, salt spray, and occasional seawater inundation, coupled with limited water and nutrient availability (Arbelet-Bonnin et al., 2019).

Concerning soil/water salinity tolerance, several studies have shown that *C. maritima* enhances growth at moderate salinity levels (50-100 mM of NaCl), exhibiting no adverse effects even up to 200 mM NaCl (Debez et al., 2004, Debez et al., 2008, Debez et al., 2012; Arbelet-Bonnin et al., 2019). The stressful conditions of its habitat elicit biochemical pathways to produce secondary metabolites as adaptive responses (Qasim et al., 2017; Corrêa et al., 2020). Secondary metabolites, including phenols, glucosinolates, sterols, vitamin C, and others, are indeed reported to be present in *C. maritima* (Mansour et al., 2018; Arbelet-Bonnin et al., 2019). These secondary metabolites exhibit powerful biological activities, including antioxidant, antimicrobial, and anti-inflammatory properties, potentially offering benefits against various human diseases (Petropoulos et al., 2018; Stanković et al., 2019).

Extracts of *C. maritima* shoot from Portugal displayed high phenolic content, antioxidant activity, and inhibitory action against key enzymes linked to human diseases (Placines et al., 2020). These findings corroborate antioxidant, anti-inflammatory, antibacterial properties, and antiproliferative effects observed in *C. maritima* extracts from France (Meot-Duros et al., 2008) and confirmed in shoot extracts against myeloma cells (Fuochi et al., 2019). These properties suggest *C. maritima* as a potential source for developing novel nutritional and functional products, addressing the growing demand for healthy foods and supplements (Petropoulos et al., 2018; Lombardi et al., 2022).

Given the expected chemical and bioactive diversity among *C. maritima* ecotypes, their evaluation is crucial for identifying those with the most promising properties. Studying their adaptive mechanisms to abiotic stress is a crucial first step in this selection process. Furthermore, research highlighting physiological differences between genotypes can contribute to understanding tolerance to harsh conditions. This knowledge can be valuable for the further exploitation of *C. maritima* in biosaline agriculture, addressing the growing interest in halophyte crops as solutions to soil degradation and salinization under climate change (Arbelet-Bonnin et al., 2019; Corrêa et al., 2020; Lombardi et al., 2022).

Limited knowledge exists on the qualitative profile and its association with physiological adaptations of Italian *C. maritima* ecotypes. While studies have explored physiological (Gratani et al., 2009) and morpho-functional adaptations (Ciccarelli et al., 2010) in natural areas of Latium and Tuscany, they have not linked these findings to the plant’s biochemical response. Notably, Ciccarelli et al. (2010) suggested a greater stress tolerance in pinnatifid lamina (PL) morphotype compared to entire lamina (EL) plants. Building upon these insights, this research aims to evaluate the potential of PL and EL morphotypes of *C. maritima* from Puglia for high-nutritional food production, therefore the morpho-physiological characteristics, bioactive compound contents, and antioxidant traits have been assessed, bridging the gap between ecophysiological adaptations and potential food applications.

## 2 Materials and methods

### 2.1 Collecting sites and samplings

**Figure 1:**
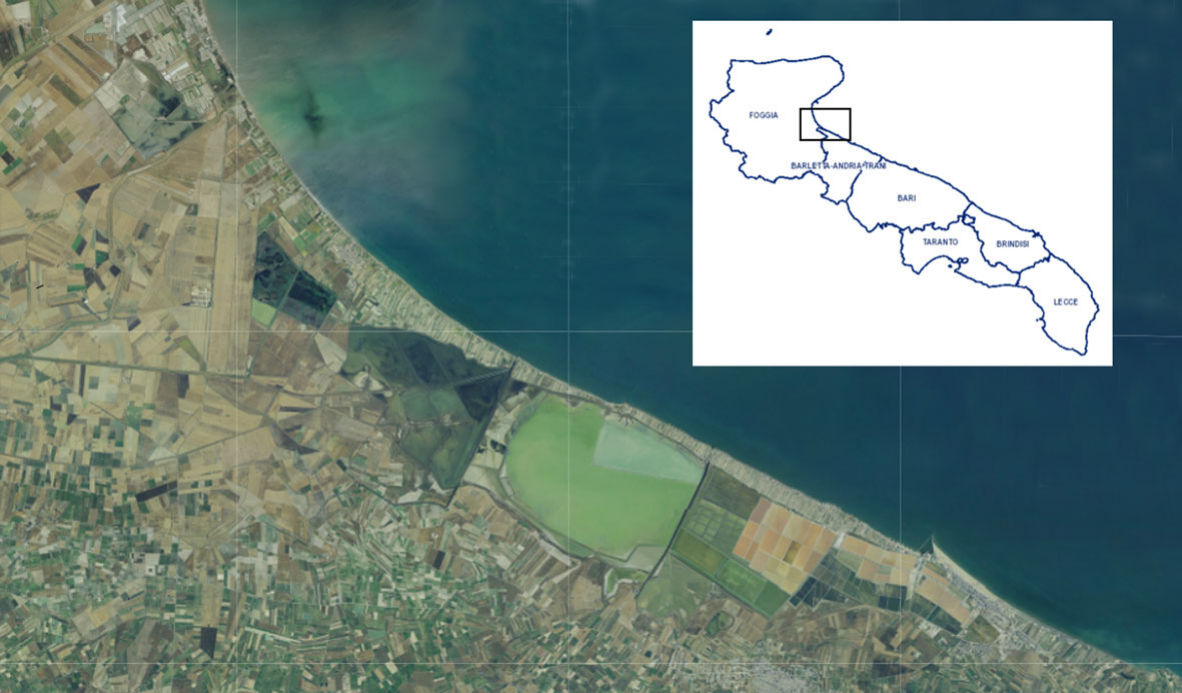
Map of the Puglia region showing the collection sites of the *Cakile maritima* morphotypes, situated between the Saltworks of Margherita di Savoia (Special Protected Area) and the Adriatic Sea.

**Figure 2:**
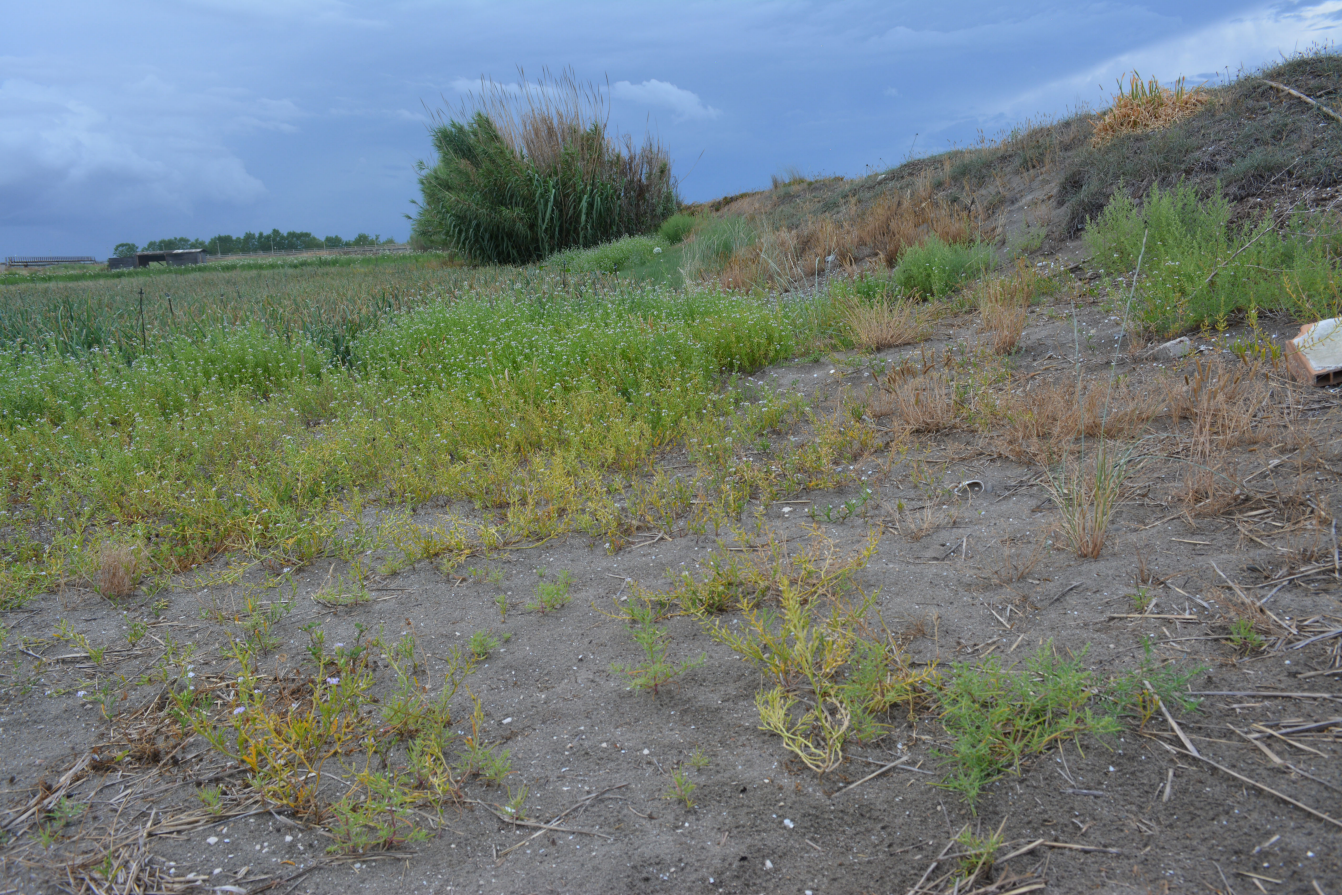
The sampling area of the *Cakile maritima* morphotypes located between the cultivated sandy fields (“*arenili*”) and the coastal dunes.

**Figure 3:**
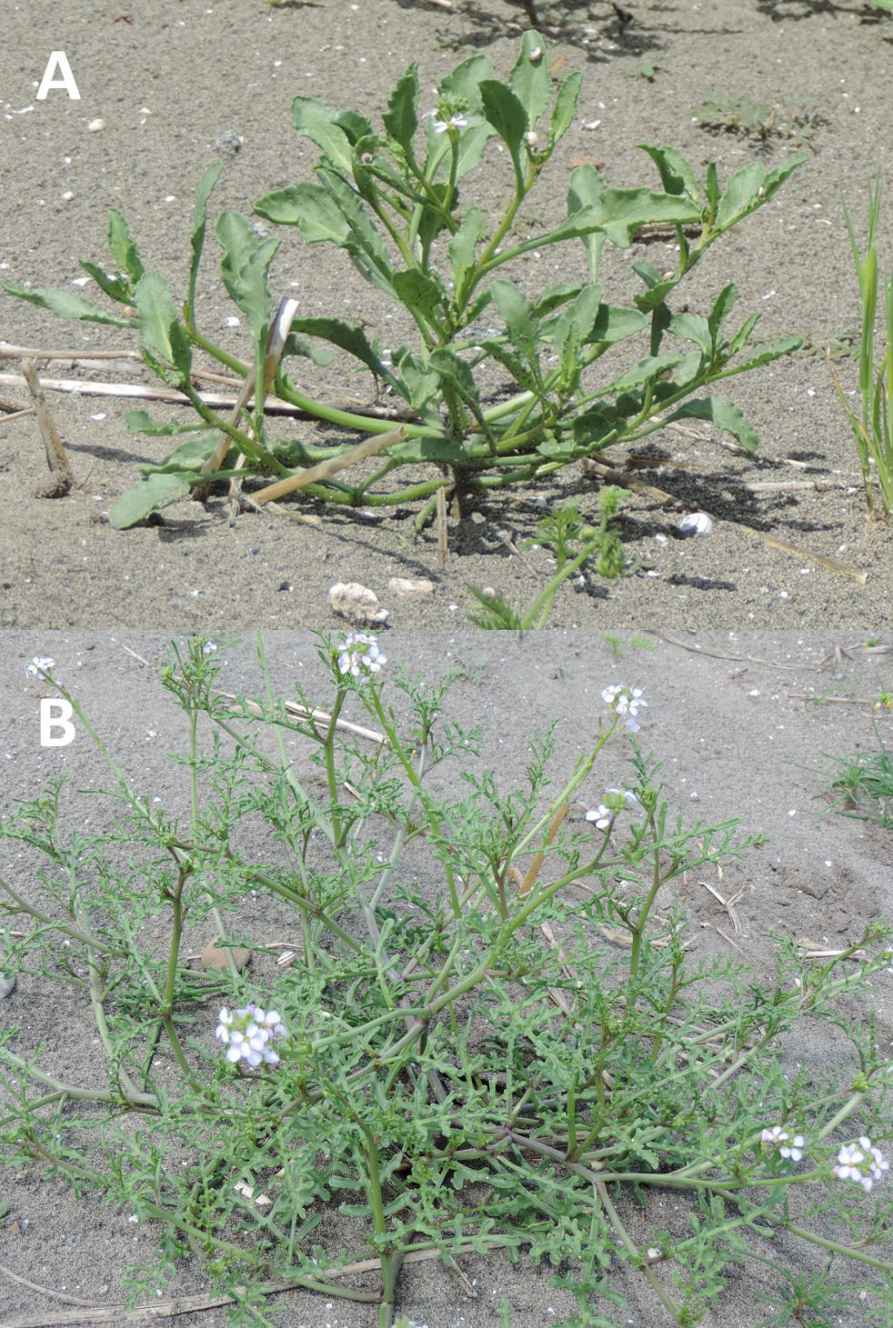
*Cakile maritima* morphotypes in their natural environment (Margherita di Savoia (BT- Italy). Plants with entire **(A)** or pinnatifid **(B)** leaves.

Plants of *Cakile maritima* were collected from an area situated between the Saltworks of Margherita di Savoia (Special Protected Area) and the Adriatic Sea, near the village Margherita di Savoia (BT), northern Puglia (latitude, 41.4° N; longitude, 16.0° E; altitude, 0-5 m a.s.l. (**Figure 1**). Specifically, sampling was conducted at three sites, each covering an area of 30 m^2^, spaced approximately 1 km apart. These sites were located between the cultivated sandy fields (“*arenili*”) and the coastal dunes (**Figure 2**). Plant materials, representing two morphotypes (entire-EL and pinnatifid-PL leaf) (**Figure 3**), at the vegetative/initial flowering stage (on March 30, 2023), characterized by succulent green stems, were randomly collected to ensure a representative sample. The species was identified by a botanist (Prof. Enrico Perrino, Department of Agriculture, Food, Natural Resources and Engineering (DAFNE), University of Foggia).

The soil of the three sampled sites is predominantly characterized by sand (96%) with low percentages of lime and clay, a pH of 7.9 and an electrical conductivity of 900 µS cm^-1^ on average. More details are reported in the **Supplementary Table 1**.

**Figure 4:**
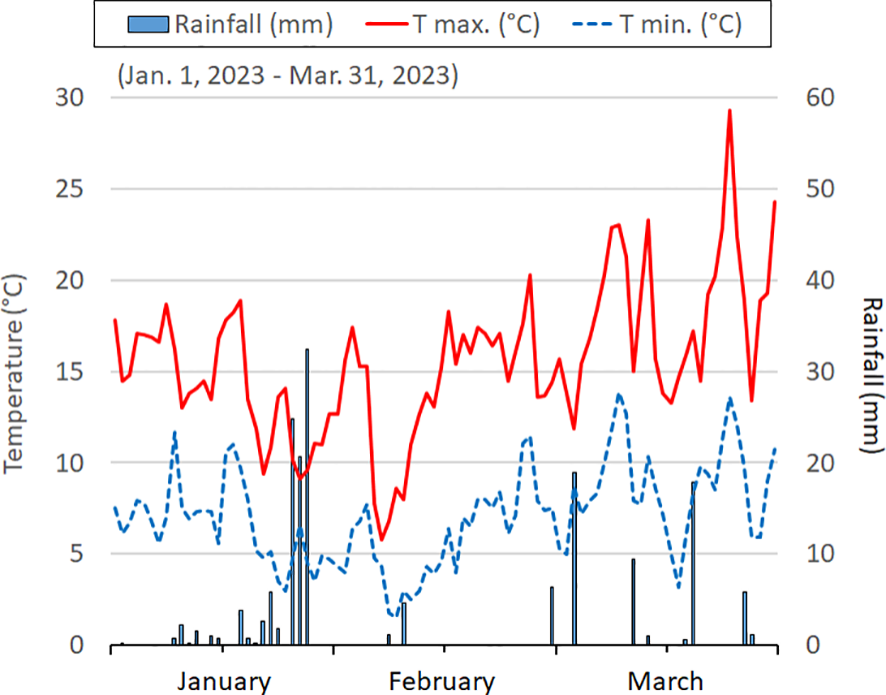
The daily temperatures (minimum and maximum) and rain events in the three months before the samplings.

The area has a Mediterranean climate characterized by mild winters and dry and warm summers. Over the past thirty years, the average minimum and maximum temperatures have been recorded at 9.8 ± 1.6 and 21.6 ± 2.1°C, respectively. The mean temperatures for the coldest and hottest months are 7.5°C (January) and 25.0°C (July), respectively. Annual rainfall averages 497 mm, with 33% occurring between October-December, 25% between January-March, 22% in the spring, and only 11% during July-August. Climatic conditions, including temperatures and rainfall, during the January-March 2023 period, are reported in **Figure 4**.

### 2.2 Plant material, bio-physiological and chemical determinations

For each site, the aerial parts (shoots) of fifteen plants representing both morphotypes were harvested. They were then divided into three subsample and subjected to freeze-drying (ScanVac CoolSafe 55-9 Pro; LaboGene ApS, Lynge, Denmark) for subsequent phytochemical analyses. They were performed in triplicate on each subsample bulk. Before freeze-drying, the fresh weight (FW) and dry weight (DW) of the shoots (dried in a thermo-ventilated oven at 70°C until they reached a constant mass) were determined for each plant of the subsamples. Additionally, the FW, DW, and leaf area (LA) measurements were taken on 5 leaves per subsample (n=45). The LA was determined by scanning the leaves at 360 dpi using an Epson Perfection V750 PRO scanner (Epson Italia s.p.a., Cinisello Balsamo, MI, Italy). The captured images were processed using the image analysis software ImageJ (National Institutes of Health, Bethesda, Maryland, USA). The dry matter concentration of shoots and leaves was calculated as (DW/FW)×100. The specific leaf area (SLA) was determined as the ratio LA/DW, while leaf mass per unit leaf area (LMA) was calculated as leaf DW/LA. Additionally, the succulence index (SI) was calculated as the ratio of the difference between DW and FW to the leaf area (Gratani et al., 2009).

#### 2.2.1 Leaf gas exchange and chlorophyll fluorescence

Field measurements were conducted on one sampling site, where leaf gas exchange and chlorophyll fluorescence measurements were taken from 8 plants for each *C. maritima* morphotype, with each plant serving as a replication. Three leaves per plant were assessed for the parameters. Leaf gas exchange parameters - net CO_2_ assimilation rate (An), intercellular CO_2_ concentration (Ci), stomatal conductance (gs) and transpiration rate (E)- along with chlorophyll fluorescence parameters - the efficiency of PSII (ΦPSII), photochemical quenching (qP) and non-photochemical quenching (qN) - were measured for *C. maritima* morphotypes. The measurements were conducted using an open circuit infrared gas exchange system equipped with a leaf fluorimeter and LED light source (Li-6400XTF, LI-COR Inc., Lincoln, NE, USA). Initially, measurements were taken under increasing light intensities (PAR from 0 to 2,500 µmol m^-2^ s^-1^ (data not shown). Subsequently, measurements were taken under specific conditions: 2,000 µmol m^−2^ s^−1^ PAR (saturating light intensity), leaf temperature maintained at 23°C, ambient CO_2_ concentration set at 400 µmol mol^−1^, and air flow rate of 300 cm^3^ s^−1^.

#### 2.2.2 Chlorophylls, carotenoids and anthocyanins

Chlorophyll concentrations, including chlorophyll *a*, *b*, and total, as well as carotenoids were determined using the spectrophotometric method described by Sumanta et al. (2014), with slight modifications. Lyophilized samples (0.05 g) were mixed with 1.5 mL 80% ethanol (ethanol:water 80:20), containing 0.1% hydrochloric acid in a 10 mL tube with a screw cap. The mixture underwent ultrasonic treatment using an ultrasonic power cleaner (DU-32 Digital; Argo Lab, Carpi, MO, Italy) at room temperature for 30 minutes. After sonication, the samples were centrifugated in a refrigerated centrifuge (Beckman Coulter AllegraTM 25, Fullerton, CA, USA) for 15 min at 4,000 g and 4°C and the supernatant was collected. This process was repeated twice and the supernatants were combined. Finally, the extract was filtered by using reinforced nylon membrane filters (0.22 µm).

The extract solution was then analyzed using a spectrophotometer (Evolution 201 UV-Visible Spectrophotometers, Thermo Scientific Waltham, MA, USA) at wavelengths of 664 and 649 nm. The following equations were used for quantification: Chl *a*=13.36(A664)-5.19 (A649); Chl *b*=27.43(A649)-8.12(A664); Chl total=17.32(A649)+7.18(A664). Carotenoids were calculated using the equation: Carotenoids=(1000(A470)-2.13Chl *a*-97.63Chl *b*)/209.

For the determination of anthocyanins, the same extract solution was utilized, following the method reported by Sims and Gamon (2002). Absorbance was measured at 650 nm and 525 nm using the spectrophotometer. To correct the interference of chlorophylls, the following equation was used: AA=A529-(0.288*A650), where AA represents the corrected anthocyanin absorbance. Total anthocyanin content was calculated as cyanidn-3-glucoside, using the corrected absorbance and a molar absorbance coefficient for anthocyanin at 525 nm of 26,900 L mol^-1^ cm^-1^ (Murray and Hackett, 1991).

#### 2.2.3 Phenols, flavonoids, glucosinolates and vitamin C

The extraction of phenols was carried out on of lyophilized sample (30 mg) with 1 mL of water/methanol (20:80 v/v) at room temperature in an ultrasonic cleaner bath (DU-32 Digital; Argo Lab, Carpi, MO, Italy) for 15 min; then the mixture was centrifuged in a refrigerated centrifuge (ThermoFisher Scientific, Waltham, MA, USA) (14,000 rpm, for 15 min, at 4°C) and the supernatant was collected. The extraction was repeated twice, and the supernatants were combined. The extracts were stored at -20°C and measured within 24 h.

Phenolic concentration was spectrometrically determined on methanolic extracts using the Folin-Ciocalteu method: 100 μL of the extracts were diluted with 3 mL of distilled water, mixed with 0.5 mL of Folin–Ciocalteu reagent and kept at room temperature for 5 min; then 1.0 mL of 20% Na_2_CO_3_ was added to the mixture. After 45 min at 30°C, absorbance was read at 750 nm (Shimatzu UV-1800, Shimadzu Scientific Instruments, North America, USA).

The values were determined from a calibration curve (0-250 mg L^-1^; R^2^ 0.998) prepared with gallic acid (g.a.) standard solutions by applying the same procedure as for the extracted sample. The results were expressed as gallic acid equivalents (g.a.e.) per unit weight.

The flavonoid extraction was carried out on lyophilized samples (30 mg) in water/methanol (1 mL) (20:80 v/v) at room temperature in ultrasonic cleaner bath (DU-32 Digital; Argo Lab, Carpi, MO, Italy) for 15 min; then the mixture was centrifuged in a refrigerated centrifuge (ThermoFisher Scientific, Waltham, MA, USA) (14,000 rpm, 15 min, 4°C) and the supernatant was collected. The extraction was repeated twice and the supernatants were combined. The extracts were stored at -20°C and analyzed within 24 h.

The determination of flavonoid content was based on the AlCl_3_ method (Chandra et al., 2014). In detail, 200 µL of the extract or diluted standard quercetin solutions were mixed with 40 µL of AlCl3 (10%), 40 µL of potassium acetate (1 M), 1,120 µL of distillate water. Samples were incubated for 30 minutes at room temperature. The absorbance was measured on UV-Vis spectrophotometry (Shimatzu UV-1800, Shimadzu Scientific Instruments, North America, USA) at 415 nm. Total flavonoids were expressed in quercetin equivalent (q.e.) per unit weight.

Glucosinolate extraction and desulphation were determined following the ISO protocol (ISO Method 9167-1, 1992).

The lyophilized sample (0.5 g) was extracted with 5 mL of 70% ethanol in a shaking water bath at 75°C for 10 min. Then the mixture was centrifuged at 15.000 g for 20 min at 4°C, and the supernatant was collected. The residue was re-extracted and the two extracts were combined and filtered. Purification of the extracts was carried out by column chromatography. Desulphation and initial separation of desulphoglucosinolates (d-GLS) were performed using mini-columns. Columns were prepared by adding anion-exchange resin (DEAE-Sephadex A-25) solubilized in acetic acid 2M to give a 0.5 mL bed volume. These columns were washed with 2 mL of 6 M imidazole formate (40 g of imidazole in 100 mL of 30% v/v formic acid), followed by two washings with 2 mL ultra-pure water. Each extract was loaded onto the pre-equilibrated columns and the unbound material was removed using 1 mL of 0.1 M sodium acetate (pH 5.8) per column, this also served to adjust the pH of the resin for the desulphation reaction. The columns were then capped and treated overnight at room temperature with 100 µL of partially purified Helix pomatia Type-1 sulfatase (EC 10.000 units) solution.

The d-GLS was eluted with two washes of 1 mL of water (and stored at -20°C if necessary) before HPLC analysis. The reference compound (sinigrin) was desulphated as the extracted GLS. The d-GLS were separated using a gradient HPLC method with an ICS 3000 System (Dionex ThermoFisher Scientific, Waltham, MA, USA) included: a gradient pump, a 10 µL injection loop, C18-5 µm reverse-phase ion-exchange columns (Kinetex Core-Shell, Phenomenex) combined with a UV-visible detector (RLSC Diode Array Detector, Dionex) set to a wavelength of 229 nm and Chromeleon version 6.50 software (ThermoFisher Scientific, Waltham, MA, USA). The oven temperature was set at 35°C. The individual compounds were separated using the following program, with a flow of 0.8 mL min^-1^: one minute at 100% H_2_O, 49 min gradient from 0% to 25% (v/v) ethanol, 4 min gradient for returning at 100% H_2_O; 10 min at 50% of acetonitrile/water (v/v); last 10 min at 100% H_2_O. Total glucosinolates was the sum of individual (unkown) glucosinulates, quantified as sinigrin equivalent.

Vitamin C content was assessed by homogenizing 0.1 g of lyophilized sample for 15 min with 5 mL of methanol/water (5:95 v/v), plus citric acid (21 g L^-1^), EDTA (0.5 g L^-1^) and NaF (0.168 g L^-1^). The homogenate was filtered through two layers of cheesecloth and the pH was adjusted to 2.2-2.4 by addition of HCl (6 mol L^−1^). After centrifugation (12,000 g, 5 min, 4°C), the supernatant was filtered through a Sep-Pak C18-cartridge (Waters, Milford, MA, USA) and then through a 0.2 μm cellulose acetate filter (INCOFAR, Modena, Italy). L-ascorbic acid (AA) and L-dehydroascorbic acid (DHAA) contents were determined as described by Zapata and Dufour (1992) with some modifications. The HPLC analysis was achieved after the derivatisation of DHAA into the fluorophore 3-(1,2-dihydroxyethyl)furol[3,4-b]quinoxaline-1-one (DFQ), with 1,2-phenylenediamine-dihydrochloride (OPDA). The extracts (20 μL) were analysed with an HPLC (AgilentTechnologies 1200 Series; Agilent, Waldbronn, Germany) equipped with a DAD detector and a binary pump. The separations of DFQ and AA were achieved on a Synergi 4u-Hydro-RP 80A C18-column (250x4.60 mm) (Phenomenex Inc., Castel Maggiore, BO). The mobile phase was methanol:water solution (5:95 v/v), containing 5 mmol L^-1^ of cetrimide and 50 mmol L^−1^ KH_2_PO_4_ at pH 4.5. The flow rate was 1 mL s^−1^. The detector wavelengths were 348 nm for DHAA and 251 nm for AA. The total vitamin C is the sum of AA and DHAA.

#### 2.2.4 Antioxidant capacity

##### 2.2.4.1 Radical scavenging capacity by the TEAC(ABTS) method

The radical scavenging capacity was assessed using a slightly modified version of the TEAC (Trolox Equivalent Antioxidant Capacity) method described by Re et al. (1999), by using the decolorization assay applied for both hydrophilic and lipophilic antioxidants. This method is based on the capacity of antioxidants to neutralize the radical cation blue/green ABTS^+•^ chromophore to a colorless ABTS form. The ABTS^+•^ form is prepared by an oxidation reaction of ABTS with potassium persulfate. A decrease in the concentration is linearly dependent on the antioxidant concentration.

The lyophilized sample (30 mg) was extracted with 1 mL of 80% methanol in DU-32 Digital ultrasonic power (Argo Lab, Carpi, MO, Italy) for 15 min. The mixture was centrifuged at 13,000 rpm for 10 min, and the supernatant was collected. The extraction was repeated twice and two supernatants were combined into a hydrophilic fraction. Subsequently, the residue was extracted twice with 1 mL of hexane in ultrasonic power for 15 min and the two supernatants were combined into lipophilic fraction. The extracts were stored at 20°C and measured within 24 h. The absorbance was measured at 734 nm after 6 min standing.

Values were determined from a calibration curve prepared with Trolox (6-hydroxy-2,5,7,8-tetramethylchroman-2-carboxylic acid) solutions prepared following the same procedure as for the extracted sample.

The results were expressed as Trolox Equivalent (T.E.) per weight unit.

##### 2.2.4.2 Radical scavenging capacity by DPPH assay

The antioxidant activity was assessed by using DPPH (2,2-diphenyl-1-picrylhydrazyl), an assay only applied for hydrophilic antioxidants (only for methanolic extracts), according to a slightly modified method reported by Lisanti et al. (2015).

This method relies on the use of the free radical DPPH. The odd electron in the DPPH free radical produces a strong absorption maximum at 517 nm and produces a color purple. The color turns from purple to yellow as the molar absorptivity of the DPPH radical at 517 nm reduces as a result of the pairing of the odd electron of DPPH radical with a proton from a free radical scavenging antioxidant to produce DPPH-H. The radical scavenging capacity was measured using a slightly modified version of the DPPH method described.

The lyophilized sample (30 mg) was extracted with 1 mL of 80% methanol in DU-32 Digital ultrasonic power (Argo Lab, Carpi, MO, Italy) for 15 min. The mixture was centrifuged at 13,000 rpm for 10 min, and the supernatant was collected. The extraction was repeated twice and two supernatants were combined into a hydrophilic fraction. The extracts were stored at 20°C and measured within 24 h.

DPPH was progressively solubilized in ethanol until a concentration producing an absorbance ranged from 1.12 to 1.08 at 517 nm. The analysis solution was obtained by mixing 1,900 µL of diluted DPPH and 100 µL of extract sample or Trolox standard solution. The absorbance was measured at 517 nm after 30 min, standing in the dark.

Values were determined from a calibration curve prepared with Trolox solutions following the same procedure as for the extracted sample.

The results were expressed as Trolox Equivalent (T.E.) per weight unit.

##### 2.2.4.3 Antioxidant activity by the FRAP method

The antioxidant activity was assessed using a slightly modified version of the FRAP (Ferric Reducing Antioxidant Power Assay) method described by Lisanti et al. (2015), an assay applied for both hydrophilic and lipophilic antioxidants.

The assay is based on the ability of the antioxidants to reduce (through an electron transfer mechanism) Fe^3+^ to Fe^2+^ ions in the presence of TPTZ (2,4,6- tripyridy-s-triazine) solution, forming an intense blue Fe^2+^–TPTZ complex with an absorption maximum at 593 nm. The reaction is pH-dependent (optimum pH 3.6) and the absorbance decrease is proportional to the content of species endowed with antioxidant activity.

The lyophilized sample (30 mg) was extracted with 1 mL of 80% methanol in DU-32 Digital ultrasonic power (Argo Lab, Carpi, MO, Italy) for 15 min. The mixture was centrifuged at 13,000 rpm for 10 min, and the supernatant was collected. The extraction was repeated twice and two supernatants were combined into a hydrophilic fraction. Subsequently, the residue was extracted twice with 1 mL of hexane in ultrasonic power for 15 min and the two supernatants were combined into lipophilic fraction. The extracts were stored at 20°C and measured within 24 h.

The FRAP reagent was prepared by combining 2.5 mL of a TPTZ solution (10 mM) in HCl (40 mM), 2.5 mL of a FeCl_3_ solution (20 mM), and 25 mL of NaOAc buffer (300 mM, pH 3.6). Then, the FRAP reagent (1.8 µL) was mixed with 200 µL of deionized water and 40 µL of the sample extracts. The reaction mixture was kept for 4 min at room temperature and successively, the corresponding absorbance was measured at 593 nm. Values were determined from a calibration curve obtained with Trolox solutions at fixed concentrations, following the same procedure as for the extracted sample.

The results were expressed as Trolox Equivalent (T.E.) per weight unit.

#### 2.2.5 Inorganic ions

Inorganic anions were extracted from the lyophilized sample (0.5 g) with 50 mL of eluent solution in a shaking water bath at room temperature for 30 min. The mixture was filtered twice through n. 2 Whatman paper and then through 0.22 µM Millipore filter, before injection into the ion chromatography system (ICS-3000, Dionex Corp., Milan, Italy). The system was equipped with an isocratic pump, a model AS-DV autosampler, a self-generating ASR anion suppressor (4 mm), A Dionex Ion-Pac AS23 (Dionex Corp., Milan, Italy) analytical column (4 mm×250 mm) and a guard column (4 mm×50 mm), maintained at 35°C. The eluent consisted of 3.5 mM sodium carbonate and 1 mM sodium bicarbonate (flow rate, 1 mL min^-1^).

Inorganic cations were extracted from lyophilized sample samples (0.3 g), previously ashed (in a muffle furnace at 550°C for 6 h), and acid digested (20 mL of 1 mol L^-1^ HCl, in boiling water for 30 min). The resulting solution was filtered through a 0.22 μm Millipore filter, diluted, and analyzed by an ion chromatography system equipped with a self-generating DRS-600 suppressor (4 mm), a Dionex IonPack CS12A analytical column (4×250 mm, 5 μm), and an eluent solution (20 mM methane-sulfonic acid) (flow rate, 1 mL min^-1^).

Cation and anion compounds were identified by a comparison of the retention times with standards. Peak areas were analyzed using Dionex Chromeleon software (version 6.80, ThermoFisher Scientific, Waltham, MA, USA)

Inorganic iodine determination was performed using the protocol by Perring et al. (2001) with some adjustments. Briefly, lyophilized samples (0.1 g) were treated with 25 mL of hot distilled water (60°C) and stirred for 30 min, at room temperature. Treated samples were rested at room temperature until cooled down. Thereafter, 25 mL of distilled water was added, samples were centrifuged at 6,000 rpm for 15 min, and filtered by using Whatman filter papers n. 2, followed by a 0.2 µm syringe membrane filter.

After the preparation and the extraction, the filtered solutions were used for quantification of inorganic iodine content by using a spectrophotometer (Shimadzu Scientific Instruments, North America, USA) at 454 nm. Iodate standard solution and the extracts samples (0.5 mL) were added with distilled water (4.5 mL) and treated with 1 mL of KSCN (2.37 mM), 2 mL of NH_4_Fe(SO_4_)_2_ 12H_2_0 (7.7% m/v) in 2.4 M HNO_3_ and 2 mL of NaNO_2_ (0.02% m/v). The solutions were mixed and incubated in a water bath at 60 ± 2°C for 1 h and subsequently incubated for 10 min in a water–ice mixture to stop the colorimetric reaction. The quantification of inorganic iodine was determined by using a calibration curve (0-12 µg L^-1^; R2 = 0.996). The accuracy of the method was verified by using NIST^®^SRM^®^1573a Tomato Leaves as certified reference material analyzed at each working session.

### 2.3 Statistical analysis

Statistical analysis was performed with the Statistical Analysis System software using the General Linear Model (GLM Proc of the SAS Software; SAS 9.1; SAS Institute, Cary, NC, USA). The mean comparison was performed using the least significant difference test (LSD) (p=0.05).

## 3 Results

### 3.1 Climatic condition

Three months before the physiological measurements and plant collections, average daily maximum temperatures ranged from 15-16°C in January-February to 17-20°C in March, with the lowest values (8°C) recorded in the first week of February. Average daily minimum temperatures were 7-8°C, except for 4-5°C recorded in January-February and 2°C for a few days (7-10 of February). Total rainfall, amounting to 167 mm, predominantly occurred in January, with sporadic events (19 and 18 mm) in March (**Figure 4**).

### 3.2 Bio-Physiological characteristics

The *C. maritima* shoots of both PL and EL morphotypes were harvested at the vegetative stage and had comparable fresh weight (FW), dry matter concentration (DM) as well as leaf DM, area and succulence index values (SI) (**Table 1**). However, PL plants showed a lower specific leaf area (SLA) and higher leaf mass area (LMA) compared with EL plants. The concentration of chlorophylls (Chl *a* and Chl *b*) did not differ significantly between the two morphotypes, with an average total chlorophyll content of 219 µg g^-1^ FW (**Table 1**).

**Table 1:** Bio-physiological traits of shoots of two morphotypes of *Cakile maritima* at the vegetative stage.

Leaf morphotype	Shoot fresh wt. (g)	Dry matter		Leaf area (cm^2^)	Specific leaf area (cm^2^ g^-1^)	Leaf mass area (mg cm^-2^)	Succulence index (mg cm^-2^)	Chlorophyll			
		Shoot	Leaf					*a*	*b*	Total
		(g kg^-1^ FW)						(µg g^-1^ FW)		
Entire - EL	14.6 a^1^ (± 2.5)	91.0 (± 5.6)	109.6 a (± 6.0)	4.7 a (± 0.7)	107.0 a (± 6.7)	9.5 b (± 0.6)	96.9 a (± 9.8)	98.6 a (± 0.9)	40.3 a (± 2.5)	133.4 a (± 4.0)
Pinnatifid - PL	16.9 a (± 5.1)	102.8 (± 5.1)	112.0 a (± 3.7)	4.4 a (± 0.5)	78.0 b (± 4.2)	13.0 a (± 0.7)	113.5 a (± 5.1)	93.5 a (± 4.5)	44.3 a (± 2.5)	134.1 a (± 6.5)
Significance^2^	ns	ns	ns	ns	**	**	ns	ns	ns	ns

^1^ Means (± standard error) in columns not sharing the same letters are significantly different according to the LSD test (p=0.05).

^2^ Significance: ** for p ≤ 0.01; ns, not significant.

Leaf gas exchange measurements revealed that the PL plants exhibited a higher net CO_2_ assimilation rate (An), stomatal conductance (gs), intercellular CO_2_ concentration (Ci), and transpiration rate (E) compared to EL plants (**Table 2**). Chlorophyll fluorescence data indicated similar values of PSII efficiency (ΦPS2) and photochemical quenching (qP), while the non-photochemical quenching (qN) were lower in the PL morphotype (**Table 2**).

**Table 2:** Photosynthetic gas exchange and chlorophyll fluorescence of two morphotypes of *Cakile maritima*.

Leaf morphotype	Photosynthetic rate (An) (μmol m^-2^ s^-1^)	Stomatal conductance to H_2_O (gs) (mol m^-2^ s^-1^)	Intercellular [CO_2_] (Ci) (μmol mol^-1^)	Transpiration rate (E) (mmol m^-2^ s^-1^)	Efficiency of PSII (ΦPSII) (-)	Photo-chemical Quencing (qP) (-)	Non-photo-chemical quencing (qN) (-)
Entire - EL	31.7 b^1^ (± 1.1)	1.0 b (± 0.1)	309.0 b (± 3.2)	15.0 b (± 0.3)	0.23 a (± 0.01)	0.36 a (± 0.04)	2.4 a (± 0.1)
Pinnatifid - PL	41.8 a (± 1.7)	1.3 a (± 0.1)	705.8 a (± 29.9)	24.2 a (± 1.0)	0.22 a (± 0.01)	0.41a (± 0.01)	2.2 b (± 0.1)
Significance^2^	***	*	***	***	ns	ns	*

^1^ Means (± standard error) in columns not sharing the same letters are significantly different according to the LSD test (p=0.05).

^2^ Significance: ***, and *, respectively, for p ≤ 0.001, and p ≤ 0.05; ns, not significant.

### 3.3 Bioactive compound contents and antioxidative capacity

The average levels of phenols, flavonoids, and glucosinolates were 172 mg gallic acid equivalents (g.a.e.) per 100 g FW, 65 mg quercetin equivalents (q.e.) per 100 g FW and 61 mg g^-1^ FW, respectively, with no significant differences observed between the PL and EL sea rocket morphotypes. However, the EL morphotype had a notably higher vitamin C level compared to the PL, despite the latter demonstrating a higher concentration of ascorbic acid. On the contrary, the concentration of carotenoids and anthocyanins was higher in the PL type (**Table 3**). Furthermore, except for the antiradical activity towards ABTS, both the DPPH and FRAP assays indicated a greater activity for the PL type (**Table 4**).

**Table 3:** Bioactive compound concentrations in shoots of two morphotypes of *Cakile maritima*.

Leaf morphotype	Dehydro-ascorbic acid	Ascorbic acid	Vitamin C	Glucosi-nulates	Carote-noids	Phenols (mg a.g.e 100 g^-1^ FW)^3^	Flavonoids (mg q.e. 100 g^-1^ FW) ^3^	Anthocyanins (mg c.g.e. 100 g^-1^ FW)^3^
	(mg 100 g^-1^ FW)							
Entire - EL	132.1 a^1^ (± 8.8)	3.1 b (± 0.3)	135.3 a (± 8.8)	54.8 a (± 7.2)	4.8 b (± 0.2)	214.1 a (± 9.7)	73.4 a (± 3.5)	3.1 b (± 0.2)
Pinnatifid - PL	68.5 b (± 7.0)	10.7 a (± 1.1)	79.2 b (± 6.9)	67.2 a (± 7.9)	5.3 a (± 0.2)	202.6 a (± 6.2)	68.7 a (± 1.8)	3.9 a (± 0.2)
Significance^2^	***	***	***	ns	*	ns	ns	**

^1^ Means (± standard error) in columns not sharing the same letters are significantly different according to the LSD test (p=0.05).

^2^ Significance: ***, **, and *, respectively, for p ≤ 0.001, p ≤ 0.01, and p ≤ 0.05; ns, not significant.

^3^ a.g.e, acid gallic equivalent; q.e., quercetin equivalent; c.g.e., cyanidin-3-glucoside equivalent.

**Table 4:** Antioxidant activity in shoots of two morphotypes of *Cakile maritima*.

Leaf morphotype	ABTS assay			FRAP assay			DPPH assay Hydrophilic
	Hydrophilic	Lipophilic	Total	Hydrophilic	Lipophilic	Total	
	(µmol T.E. g^-1^ FW)^3^						
Entire - EL	12.4 a^1^ (± 0.5)	0.88 a (± 0.03)	13.2 a (± 0.5)	3.1 b (± 0.2)	0.08 a (± 0.01)	3.2 b (± 0.23)	7.6 b (± 0.5)
Pinnatifid - PL	11.4 b (± 0.3)	0.86 a (± 0.03)	12.2 a (± 0.4)	3.6 a (± 0.1)	0.06 b (± 0.01)	3.7 a (± 0.1)	10.7 a (± 0.4)
Significance^2^	ns	ns	ns	**	***	**	***

^1^ Means (± standard error) in columns not sharing the same letters are significantly different according to the LSD test (p=0.05).

^2^ Significance: ***, and **, respectively, for p ≤ 0.001, and p ≤ 0.01; ns, not significant.

^3^ T.E., trolox equivalent.

### 3.4 Inorganic cation and anion concentrations

Regarding the main inorganic ion concentrations, cations such as K, Ca, Mg and Na were found at a higher level in the EL type, except for ammonium (NH_4_). On the contrary, the EL morphotype of *C. maritima* exhibited lower concentrations of nitrate, chloride, and sulphate, with no differences in iodine and phosphate levels (**Table 5**). The oxalate concentration was below the detectable level in both morphotypes.

**Table 5:** Inorganic anion and cation concentrations in shoots of two morphotypes of *Cakile maritima*.

Leaf morphotype	Cl	NO_3_	PO_4_	SO_4_	Na	K	Ca	Mg	NH_4_	I
	(mg kg^-1^ FW)									(µg 100 g^-1^ FW)
Entire - EL	2,369 b^1^ (± 93)	856 b (± 28.3)	5945 a (± 34.5)	2,227 b (± 92)	4,382 a (± 496)	7,377 a (± 299)	7,413 a (± 435)	1,459 a (± 148)	7.7 a (± 0.9)	73.6 a (± 1.6)
Pinnatifid - PL	2,802 a (± 68)	1,095 a (± 52)	747 a (± 25.5)	2,413 a (± 84)	2,659 b (± 114)	5,993 b (± 322)	5,773 b (± 294)	1,004 b (± 46)	8.5 a (± 0.5)	88.0 a (± 4.8)
Significance^2^	*	***	ns	*	**	**	**	**	ns	ns

^1^ Means (± standard error) in columns not sharing the same letters are significantly different according to the LSD test.

^2^ Significance: ***, **, and *, respectively, for p ≤ 0.001, p ≤ 0.01, and p ≤ 0.05; ns, not significant.

## 4 Discussion

### 4.1 Eco-physiological traits

Leaf morphology serves as a valuable indicator for discerning the adaptation of *C. maritima* to its natural habitats. Previous studies, conducted on ecotypes from the coastal areas of Tuscany (Italy) (Ciccarelli et al., 2010), noted differences in bio-anatomical traits between plants with pinnatifid and entire lamina types. These differences, such as leaf dorsiventral roll, larger epidermal vesicular cells and increased essential oil content in stomata, were associated with enhanced adaptability to high temperatures, solar radiation and drought of the PL type. Notably, specific leaf area (SLA) was reported to be reduced in the PL type, indicating a decreased leaf expansion compared to the EL type for the same dry mass unit, with resource allocation towards the C structural pool (cell walls, composed of cellulose, hemicellulose or lignin). This response has been linked to improved leaf defence and lifespan under resource-poor environments (Ciccarelli et al., 2010) including drought stress (Gratani et al., 2009). Further studies, evaluating the adaption of *C. maritima* and other halophytes in coastal dunes of the Latium region (Italy), highlighted that a higher leaf mass area (LMA), which is the inverse of SLA, may be indicative of greater plant adaptability to such habitat (Gratani et al., 2009).

In our study, lower SLA (and higher LMA) in PL sea rocket was confirmed (**Table 1**), suggesting that the pinnatifid morphotype may be more adaptable to tolerate the challenging conditions of their habitat. The specific abiotic stresses in this habitat are likely associated with the extremely limited water availability (only 37 mm of rainfall in March) (**Figure 4**), along with the observed low content of N, K and micronutrients in soils.

Considering the measured electrical conductivity, sodium concentration, and low percentage of cation exchange capacity (CEC) saturation by Na in the soil (**Supplementary Table 1**), it seems unlikely that salinity stress at the root level can be a significant factor, although exposure to marine aerosols occurred. However, shoot Na concentration observed in both PL (1.1 mmol g^-1^ DW) and EL (2.0 mmol g^-1^ DW) morphotypes was similar to that reported for non-NaCl-treated plants of *C. maritima* in previous studies (Debez et al., 2008; Ellouzi et al., 2011; Hamed-Louati et al., 2016; Amor et al., 2020). Furthermore, regardless of morphotype, the K/Na ratio was close to 2 (**Table 5**), indicating that competition between Na for K, which typically occurs under high-salinity conditions (Debez et al., 2013; Agudelo et al., 2021) was negligible.

The net photosynthetic rate (An) observed in PL sea rocket is consistent with the hypothesis of greater tolerance of this genotype to environmental stresses, as it displayed improved CO_2_ fixation, due to a higher stomatal conductance (gs) and stomatal CO_2_ concentration (Ci) (**Table 2**). This evidence is further supported by the analysis of chlorophyll *a* fluorescence (**Table 2**).

Both morphotypes showed similar effective efficiency of the PSII (ΦPSII) and the photochemical quenching (qP), which indicates the portion of light used for photochemistry activity. Despite the similarity in ΦPSII and qP between PL and EL morphotypes, the EL morphotype exhibited a higher energy allocation towards non-photochemical processes (qN) (**Table 2**).

Under stressful conditions such as cold, drought, nutrient deficiency (Ashikhmin et al., 2023), salinity (Debez et al., 2012) or fluctuating light (Malnoë, 2018; Shi et al., 2022), plants may absorb light energy beyond their capacity to utilize it in photosynthesis. This can lead to photo-inhibition, a process involving damage to the photosynthetic apparatus, particularly PSII, resulting in the formation of reactive oxygen species (ROS) and a decrease in photosynthetic efficiency (Ashikhmin et al., 2023).

Non-photochemical quenching (qN) serves as a mechanism to prevent photo-inhibition by dissipating excess energy as heat. It involves various processes at the chloroplast level, such as the violaxanthin-zeaxanthin cycle (Malnoë, 2018). However, photoprotection mechanisms can vary between plants, due to differences in pigment levels. Specifically, carotenoids adsorb short-wavelength light and transfer energy to chlorophylls, thereby preventing the formation of chlorophyll triplets and ROS. Anthocyanins effectively screen visible light, thereby preventing ROS production induced by excessive light. Furthermore, both carotenoids and anthocyanins may act as antioxidant compounds, directly scavaging ROS, along with other antioxidants such as vitamin C, phenols, flavonoids and glutathione. Thus, they represent additional crucial mechanisms of photoprotection (Ashikhmin et al., 2023).

In the current study, photoprotection mechanisms present in the EL morphotype arguably appear to involve higher levels of energy dissipation (qN) and scavenging of reactive oxygen species, which can be linked to the antioxidative action of the ascorbic/dehydroascorbic acid (AA/DHAA) pool. AA is the main biologically active form of vitamin C, as it interacts with ROS undergoing oxidation to DHAA; in plants, DHAA is either recycled to AA by the dehydro-ascorbate reductase (DHAR) or irreversibly hydrolysed (Gallie, 2013).

In the present research, the elevated levels of vitamin C observed in EL sea rocket were predominantly represented by DHAA (DHAA/AA=44) (**Table 3**), indicating a robust reducing action carried out by AA. In contrast, the photoprotective mechanisms in PL sea rocket appear to be more closely associated with a higher level of both carotenoids and anthocyanins (**Table 3**), as well as overall greater antioxidant capacity, as indicated by FRAP and DPPH tests (**Table 4**). The latter findings are consistent with previous studies on *C. maritima* reporting an increase in carotenoids when grown at high temperatures in natural environments (Gratani et al., 2009), and an increase in anthocyanins when grown under saline (Debez et al., 2008, Debez et al., 2012; Mansour et al., 2018; Farhat et al., 2021) or K-starvation (Houmani et al., 2022) conditions. Notably, studies involving anthocyanins were predominantly conducted on pinnatifid sea rocket as can be inferred from the pictures in the cited paper.

Thus, a notable disparity between the two morphotypes emerges in their mechanisms for contrasting light-induced damage, which in EL type primarily involve non-photochemistry quenching (qN) processes. These processes encompass photoprotective mechanisms and photo-inhibitory quenching (qI), which corresponds to a reduction in the quantum yield of photosynthetic carbon fixation (Malnoë, 2018). Given these insights, the markedly lower leaf CO_2_ fixation observed in EL plants (**Table 2**) suggests a greater contribution of photo-inhibition to qN. This hypothesis is further supported by evidence indicating reduced recycling of AA in the EL morphotype. A rise in qI and ROS levels, along with a decrease in CO_2_ assimilation, has been associated with a decline in AA recycling, due to reduced DHAR expression of DHAR (Gallie, 2013). In agreement with our findings, comparative studies on plant phenotypes/genotypes have underlined changes in the extent and composition of qN related to plant ecology (Maxwell and Johnson, 2000).

Moreover, in a natural environment characterized by abiotic stresses, a discrepancy often exists between the efficiency of PSII photochemistry (ΦPSII) and the efficiency of CO_2_ fixation (An), primarily attributed to the occurrence of photorespiration (Maxwell and Johnson, 2000), addressed for protecting PSII and sustaining photosynthesis (Shi et al., 2022). In our study, the more pronounced reduction in the rate of CO_2_ assimilation (An) in the EL morphotype is likely due to a higher contribution of photorespiration. Elevated levels of photorespiration were associated with a decrease in stomatal conductance (gs), as observed in Kangasjärvi et al., 2012, and our study concerning the EL morphotype (**Table 2**). In contrast, anatomical features of the PL morphotype, such as larger and outwardly turned epidermal vesicular cells, along with cell guards exhibiting a richer content of essential oils, suggest enhanced protection against solar radiation and thermal stress (Ciccarelli et al., 2010). These leaf characteristics likely enable the PL genotype to sustain higher transpiration rates and keep stomata open for longer periods, thereby facilitating increased CO_2_ intake (Ci, **Table 2**). All these findings may elucidate the higher frequency of the PL morphotype compared to EL in the study area (personal communication), as also reported by Ciccarelli et al. (2010) in more disturbed sites.

### 4.2 Nutritional and antioxidative traits

#### 4.2.1 Bioactive compounds and antioxidant activity

Vegetables are rich in bioactive compounds, that provide desirable health-beneficial effects beyond basic nutrition, with positive implications for human health related to antioxidant activity. These bioactive compounds, mainly secondary metabolites, play pivotal roles in combating both abiotic stressors (light, temperature fluctuations, and nutrient availability) as well as biotic stresses (insect infestations and pathogen attacks) (Cartea et al., 2011; Barba et al., 2016). Brassicaceae vegetables, in particular, represent a good source of natural antioxidants, mainly due to the presence of phenolic compounds, as well as to high levels of carotenoids and ascorbic acid (Cartea et al., 2011). Epidemiological studies underscore the protective role of these compounds against a spectrum of human diseases, including cardiovascular ailments, cancers, and neurodegenerative conditions. Carotenoids, notably, play a pivotal role in fortifying the immune system (Davey et al., 2000; Gutiérrez-Grijalva et al., 2016; Ahmad et al., 2019). Furthermore, glucosinolates (GLSs), predominantly found in the Brassicaceae family, exert a substantial role in human nutrition. Upon disruption of plant cellular membranes, these compounds are hydrolysed to various bioactive breakdown products such as isothiocyanates, nitriles and thiocyanates, which have been linked to the anticarcinogenic properties of Brassica vegetables (Cartea and Velasco, 2008).

Among bioactive compounds, a distinctive content of vitamin C has been observed for the EL morphotype, exhibiting a higher prevalence for the DHAA form (**Table 3**). PL sea rocket, displayed a halved vitamin C content, albeit with a confirmed high DHAA/AA ratio (6.1) (**Table 3**). In agreement with our findings (**Table 3**), previous work carried out on Tunisian sea rocket, cultivated under greenhouse conditions, highlighted an elevated DHAA/AA ratio, particularly under salinity stress (Mansour et al., 2018). The Authors also reported a substantially higher content of vitamin C (close to 400 mg 100 g^-1^ FW in non salt-stressed plants) compared to our plants (**Table 3**). However, the EL morphotype exhibited a vitamin C concentration (**Table 3**) very similar to that reported by Guil-Guerrero et al. (1999) (178 mg 100 g^-1^ FW) for Spanish ecotypes and comparable to or higher than other halophytes (Hulkko et al., 2022). Conversely, studies conducted on wild and cultivated sea rocket, across diverse habitats in Egypt, reported an average vitamin C content of 61.2 mg 100 g^-1^ FW (Azim and Yaecob, 2009), in line with the value found in PL plants (**Table 3**).

According to data reported by Davey et al. (2000) and the classification by the Italian National Centre of Agriculture (INRAN-CREA (Centro di Ricerca per l’Agricoltura e l’Analisi dell’Economia Agraria), 2024), sea rocket can be classified among the richest sources of vitamin C, similar to some leafy (parsley, wild rocket, rapini), and fruit vegetables (e.g. peppers). From a nutritional point of view, no concerns arise from the consumption of DHAA as it can be readily reduced to AA by the human body (Davey et al., 2000).

It is noteworthy that the assumption of 100 g of raw EL shoots (**Table 3**) could potentially exceed 100% of the recommended vitamin C daily allowance for adults (60-75 mg).

Neither glucosinolates nor phenol compounds exhibited variations according to morphotypes, suggesting a similar response of PL and EL plants to environmental factors triggering the biosynthesis of these metabolites.

Limited research has been conducted on glucosinulates in *C. maritima*. Along with leaf and fruit morphology, glucosinolate profiles have been proposed as valuable taxonomic markers for *C. maritima* with discernible differences in the glucosinolate profile of seeds between subspecies (11 compounds; Davy et al., 2006). Radwan and Soliman (2008) identified 4 compounds in shoots of *Cakile maritima* from Egypt, without providing the quantitative data. Our findings revealed higher glucosinulate levels (1,877 µmol kg^-1^ FW) compared to rapini and especially turnip tops grown in the same area (northern Puglia) under open-field conditions (Conversa et al., 2016; Conversa et al., 2020b). Nevertheless, more in-depth studies deserve the qualitative characterization of glucosinolate of the species, which could elucidate genotype-based differences.

The average phenolic content of *C. maritima* in our study (209 mg 100 g^-1^ FW; 1,855 mg 100 g^-1^ DW) surpassed that of the Tunisian ecotypes greenhouse-grown under both saline and non-saline conditions and monitored at the vegetative and flowering stage (605 mg 100 g^-1^ DW, on average) (Mansour et al., 2018). Similarly, a lower phenolic content was observed in an ecotype from Spain, grown in a controlled environmental chamber (517 mg 100 g^-1^ DW) (Agudelo et al., 2021). On the contrary, a higher level of phenols has been reported in other Tunisian ecotypes, grown in natural habitats (500-700 mg 100 g^-1^ FW) (Ksouri et al., 2008) or controlled environment (close to 4,302 mg 100 g^-1^ DW) (Ksouri et al., 2007). Additionally, ecotypes from Brittany (France) exhibited a higher content of phenols (2,224 mg 100 g^-1^ DW) (Meot-Duros et al., 2008).

In comparison to leafy or inflorescence vegetables cultivated in open fields within the same area, a significantly lower phenolic level was observed in turnip tops (about 30 mg per 100 g FW) (Conversa et al., 2020b), rapini (Conversa et al., 2016), and lettuce (about 4 mg per 100 g FW) (Bonasia et al., 2013), while spinach exhibited similar levels (Conversa et al., 2014).

Within the array of coloured flavonoids, anthocyanins stand out as the most important group of pigments, not only linked to photosynthetic machinery as previously discussed but also due to their notable biological properties (Cartea et al., 2011). In our study (**Table 3**), these pigments were detected in greater abundance compared to other ecotypes (0.1-0.4 mg 100 g^-1^ FW) (Mansour et al., 2018; Farhat et al., 2021; Houmani et al., 2022). Similarly, levels of carotenoids (**Table 3**) were comparable to those reported for other sea rocket ecotypes (Guil-Guerrero et al., 1999; Mansour et al., 2018) and other halophytes (Hulkko et al., 2022).

The variability in phenols, including flavonoids and anthocyanins, can be substantially explained by considering both genotype and environmental factors (Ksouri et al., 2007, Ksouri et al., 2008; Stanković et al., 2019), underlining the significance of characterizing local ecotypes for their nutritional valorisation (Petropoulos et al., 2018).

Among the antioxidant assays conducted, both FRAP and DPPH underlined an enhanced reductive status for PL shoots (**Table 5**), mainly attributed to the increased content of ascorbic acid, anthocyanins, carotenoids and other still undetected antioxidant compounds such as condensed tannins (Qasim et al., 2017).

#### 4.2.2 Macro and micro nutrients

Further divergence between PL and EL sea rocket morphotypes was observed in macro- and meso-nutrient concentrations, with the EL variant exhibiting enhanced levels of detected cations and lower concentrations of nitrate, chloride, and sulphate (**Table 5**). Among the cations, ammonium was detected in both morphotypes, consistent with findings reported by Davy et al. (2006). The cumulative amount of cations exceeds that of anions. Since oxalates were not detected, it is conceivable that organic acids may have balanced cations, however, these compounds were not measured in our study.

The physiological mechanisms underlying these findings and the extent to which observed changes in anion and cation uptake may represent an adaptive response to the environment (Mulet et al., 2020) proved challenging to elucidate in this study. This part of the research aimed to highlight the nutritional characteristics of these sea rocket ecotypes for potential food utilization. Therefore, a more comprehensive approach is needed to explore differences between the two sea rockets in ion uptake and homeostasis, as well as the potential influence of abiotic stresses.

Only a limited number of specific studies investigating the mineral nutrient composition of sea rocket have been conducted. Guil-Guerrero et al. (1999), examining various wild edible brassicas from natural areas of Murcia (Spain), pointed out that *C. maritima* exhibited a Na level of 308 mg 100 g^-1^ FW, a value closely resembling the mean observed in our study. However, K, Ca and Mg concentrations were notably lower (293, 39, and 77 mg 100 g^-1^ FW, respectively). In another study, nutritional assessments of three hydroponically grown halophytes were undertaken to assess their response to increasing NaCl levels (Agudelo et al., 2021). Among these halophytes, at NaCl=0 mM in the nutrient solution, *C. maritima* exhibited approximately 2,300, 2,940, 787, and 117 mg 100 g^-1^ DW of Na, K, Ca, and Mg, respectively. Also compared to the results of Agudelo et al. (2021), the amounts of K, Ca and Mg in our plants were much higher (close to 6,000, 6,960 and 1,090 mg 100 g^-1^ DW, respectively), whereas the Na level was comparable (2,384 and 3,887 mg 100 g^-1^ DW in the PL and EL morphotype, respectively). The latter evidence confirms that the plants in our study were grown under non-saline conditions.

Irrespective of genotypic variations, the K, Ca and Mg contents in sea rocket far surpass those found in common vegetables and vegetable products across European countries, rather mirroring herbs and spices such as purslane, angelica, thyme, sage, common nettle, dill leaves (EFSA, 2024).

Iodine, a trace mineral essential for human health, plays a crucial role in thyroid hormone production, metabolic regulation and brain growth (Sorrenti et al., 2021). The recommended dietary allowance (RDA) for iodine stands at 150 μg per day for adults (EFSA, 2014). Despite the absence of scientific reports on iodine concentration in sea rocket, several halophytes collected from saline areas, such as salt pans, have shown iodine levels close to (e.g., *Atriplex halimus*, *Inula crithmoides*, *Sarcocornia fruticosa*) or even higher than (*Suaeda vera*, *Beta maritima*) (Duarte et al., 2022) those observed in the present study for *C. maritima* (84 µg 100 g^-1^ FW, on average) (**Table 5**).

The mean iodine concentration observed in sea rocket was approximately 42 times higher than that reported for leafy vegetables (2.3 µg 100 g^-1^ FW; Haldimann et al., 2005), and even surpassed levels found in the iodine-biofortified leafy Brassica (66 µg 100 g^-1^ FW; Gonnella et al., 2019).

Consuming 100 g of fresh sea rocket shoots could provide nearly 56% of the RDA for iodine. Furthermore, considering its potential use as “green salt” (Cardoso et al., 2021), 51 µg of dehydrated powdered shoots could fulfil the RDA for this essential micronutrient.

One of the primary concerns regarding the utilization of halophytes for food consumption is related to the potential accumulation of significant quantities of Na and Cl in edible portions, which could contribute to pathologies such as hypertension and cardiovascular diseases (Burnier et al., 2015). The World Health Organization (WHO, 2012) and the European Food Safety Authority (EFSA, 2019) recommend a maximum daily sodium intake of 2.0 g. Furthermore, maintaining a low Na/K ratio in food is advised to mitigate the risk of high blood pressure and stroke incidence (Park et al., 2016). In our study, both sea rocket morphotypes had Na/K close to 0.5, indicating that the increased accumulation of Na observed in the EL shoots was consistent with K levels (**Table 5**). Therefore, the Na level of these ecotypes does not pose a risk to human health.

Contrary to the findings of Guil-Guerrero et al. (1999), our results revealed an oxalate content below detectable levels, suggesting no impairment of calcium bio-availability for human consumption.

While nitrate intake may potentially benefit human metabolism by reducing blood pressure (Jonvik et al., 2016) or serving as an ergogenic substance (Macuh and Knap, 2021), excessive nitrate consumption is considered detrimental to human health. To address this concern, the European Commission has established maximum nitrate levels in various foods, including leafy species (EU 915/2023), with the European Commission’s Scientific Committee on Food (SCF) and the WHO setting the allowed daily intake (ADI) at 3.7 mg kg^-1^ of body weight (Santamaria, 2006).

Our sea rocket samples fall within the ‘medium’ category (<1,000 mg kg^-1^ FW) according to the classification proposed for vegetables based on their nitrate levels (Santamaria, 2006). Their nitrate concentration is considerably lower than both cultivated (Santamaria, 2006) and wild (Bianco et al., 1998) leafy species and well below the maximum nitrate levels set by the EU 915/2023 for leafy species.

## 5 Conclusions

This study provides the first comprehensive analysis of an Italian ecotype of *C. maritima* subsp. *maritima*, evaluating both its ecophysiological traits and nutritional characteristics. Notably, the study included plants with distinct leaf morphologies [entire lamina (EL) and pinnatifid lamina (PL)]. Our findings suggest that *C. maritima* shoots hold promise as a high-nutritional food source, due to their content of bioactive compounds like phenols, glucosinolates, and vitamin C. Additionally, the mineral profile is noteworthy, particularly its high levels of cations (K, Ca, Mg), and iodine.

The observed differences in chemical composition between EL and PL plants offer the potential for enhancing the nutritional value of food products. EL plants exhibit higher concentrations of K, Ca, Mg, and vitamin C, while PL plants show superior antioxidant activity, imputable to carotenoids and anthocyanins, though further studies are needed to identify ulterior potential contributors to antioxidant activity. Additionally, the profiling of phenols and glucosinolates deserves further consideration to nutritionally distinguish between the two morphotypes, and shed light on their organoleptic characteristics (taste, flavour).

This research unveils physiological distinctions between *C. maritima* morphotypes, linking them to morphological and chemical features. Notably, PL plants appear to be more adapted to water/nutrient limitations and the coastal climate of southern Italy. Nevertheless, further investigations, including genetic characterization, are needed to validate the observed divergences in the adaptation strategies against environmental stresses of PL and EL plants, also aimed at shedding light on its tolerance to salinity.

These studies are crucial to assess their potential cultivation as a novel vegetable crop in marginal areas and/or intensive cropping systems (greenhouses, indoor/soilless systems) with applications in producing innovative food products like microgreens and baby leaf vegetables.

## Data availability statement

The raw data supporting the conclusions of this article will be made available by the authors, without undue reservation.

## Author contributions

GC: Funding acquisition, Conceptualization, Methodology, Project administration, Formal analysis, Writing – original draft, Writing – review & editing. LB: Investigation, Writing – original draft. CL: Investigation, Writing – original draft. AB: Investigation, Writing – original draft. AE: Writing – review & editing, Visualization.
