# Supplementary figures and images for: Ecophysiological and nutritional characterisation of two morphotypes of Cakile maritima subsp. maritima Scop. from Puglia region, Southern Italy
Source: Front Plant Sci. 2024 Jun 14;15:1397852. doi: 10.3389/fpls.2024.1397852 (PMC11211591; doi:10.3389/fpls.2024.1397852)

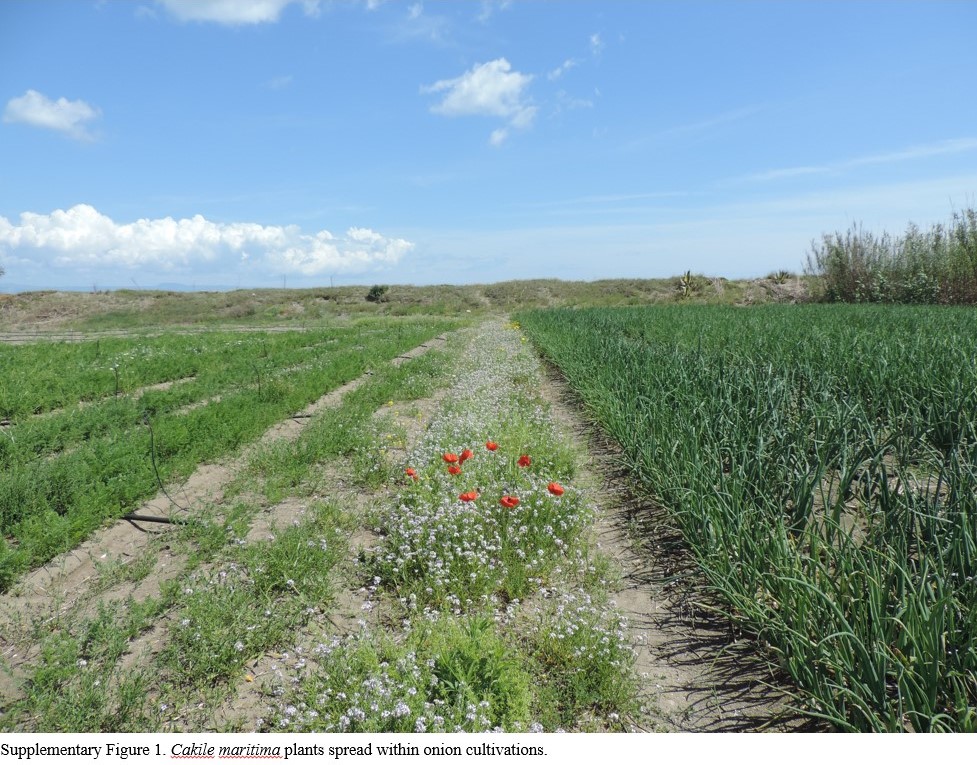

Supplement: Supplementary file 1 [file Image_1.jpeg]
