# Supplementary material for: Ecophysiological and nutritional characterisation of two morphotypes of Cakile maritima subsp. maritima Scop. from Puglia region, Southern Italy
Source: Front Plant Sci. 2024 Jun 14;15:1397852. doi: 10.3389/fpls.2024.1397852 (PMC11211591; doi:10.3389/fpls.2024.1397852)
Supplement: Supplementary file 2 [file Table_1.docx]

Supplementary Table 1- Physic-chemical characteristics of soils of the three sites where *Cakile maritima* plants were collected. The sites were located between the cultivated sandy fields (“*arenili*”) and the coastal dunes of Margherita di Savoia (BT) village, northern Puglia.

|  |  | **Sampled sites** | | | | |
| --- | --- | --- | --- | --- | --- | --- |
| **Determinations** | **Units** | **Site 1** | | | **Site 2** | **Site 3** |
| Sand | % | | 94.8 | 96.1 | | 97.5 |
| Lime | % | | 0 | 0 | | 0 |
| Clay | % | | 5.2 | 3.9 | | 2.5 |
| pH |  | | 7.9 | 7.9 | | 7.9 |
| EC | µS cm^-1^ | | 1090 | 921.5 | | 838 |
| Organic matter | g 100 g^-1^ | | 0.5 | 0.6 | | 1.0 |
| Cation exchange capacity | mmol kg^-1^ | | 24 | 40 | | 37 |
| Total nitrogen | g kg^-1^ | | 0.48 | 0.31 | | 0.34 |
| P plant-available | mg kg^-1^ | | 3.7 | 3.2 | | 3.9 |
| K plant-available | mg kg^-1^ | | 21.0 | 22.0 | | 18.6 |
| Na plant-available | mg kg^-1^ | | 48 | 45 | | 46 |
| Fe plant-available | µg kg^-1^ | | <2010 | <2010 | | <2010 |
| Zn plant-available | µg kg^-1^ | | <100 | <100 | | <100 |
| Mn plant-available | µg kg^-1^ | | 470 | 590 | | 630 |
| Cu plant-available | µg kg^-1^ | | 33 | 30 | | 36 |
| Mo plant-available | µg kg^-1^ | | 3 | 3 | | 3 |
